# Toxicity Testing in the 21^st^ Century Beyond Environmental Chemicals

**DOI:** 10.14573/altex.1506201

**Published:** 2015

**Authors:** Costanza Rovida, Shoji Asakura, Mardas Daneshian, Hana Hofman-Huether, Marcel Leist, Leo Meunier, David Reif, Anna Rossi, Markus Schmutz, Jean-Pierre Valentin, Joanne Zurlo, Thomas Hartung

**Affiliations:** 1 CAAT-Europe, University of Konstanz, Konstanz, Germany; 2 Tsukuba Drug Safety, Biopharmaceutical Assessment Core Function Unit, Eisai Co., Ltd., Ibaraki, Japan; 3 Eurofins BioPharma Product Testing Munich GmbH, Planegg, Germany; 4 Danone Food Safety Center, Utrecht, The Netherlands; 5 Bioinformatics Research Center, Department of Biological Sciences, North Carolina State University, Raleigh, NC, USA; 6 European Food Safety Authority (EFSA), Parma, Italy; 7 Consultant, Basel, Switzerland; 8 Non-Clinical Development, UCB-Biopharma, Belgium; 9 CAAT, Johns Hopkins University Bloomberg School of Public Health, Baltimore, MD, US

**Keywords:** food ingredients, drugs, Tox21c, safety assessment

## Abstract

After the publication of the report titled *Toxicity Testing in the 21^st^ Century – A Vision and a Strategy*, many initiatives started to foster a major paradigm shift for toxicity testing – from apical endpoints in animal-based tests to mechanistic endpoints through delineation of pathways of toxicity (PoT) in human cell based systems. The US EPA has funded an important project to develop new high throughput technologies based on human cell based *in vitro* technologies. These methods are currently being incorporated into the chemical risk assessment process. In the pharmaceutical industry, the efficacy and toxicity of new drugs are evaluated during preclinical investigations that include drug metabolism, pharmacokinetics, pharmacodynamics and safety toxicology studies. The results of these studies are analyzed and extrapolated to predict efficacy and potential adverse effects in humans. However, due to the high failure rate of drugs during the clinical phases, a new approach for a more predictive assessment of drugs both in terms of efficacy and adverse effects is getting urgent. The food industry faces the challenge of assessing novel foods and food ingredients for the general population, while using animal safety testing for extrapolation purposes is often of limited relevance. The question is whether the latest paradigm shift proposed by the Tox21c report for chemicals may provide a useful tool to improve the risk assessment approach also for drugs and food ingredients.

## 1 Introduction

In 2004, the US Environmental Protection Agency (EPA) requested that the National Academy of Sciences review existing strategies and develop a vision for the future of toxicity testing. A committee comprising 22 experts in various fields of toxicology, epidemiology, environmental health, risk assessment and animal welfare representing academia, industry and non-governmental organizations worked together for four years and produced its ultimate report titled *Toxicity Testing in the 21^st^ Century – A Vision and a Strategy* (Tox21c) (NRC, 2007). In this report, the committee proposed a major paradigm shift for toxicity testing – from apical endpoints in animal-based tests to mechanistic endpoints through delineation of pathways of toxicity (PoT) in human-based cell systems.

Closely linked to the report, EPA spent more than $100 million in a top-down activity in anticipation of the possible reauthorization of TSCA (Toxic Substances Control Act): high-through-put technologies used and developed mainly by pharmaceutical industries were adapted to environmental chemicals and supplemented with valuable and relevant compounds from pharmaceutical industry. This ToxCast program was also expanded with NIH (National Institutes of Health) and FDA (Food and Drug Administration), forming the Tox21^™^ alliance to an enlarged substance testing but with fewer biological assays. These methods are currently being incorporated into the chemical risk assessment process in the US (Krewski et al., 2014).

In the pharmaceutical industry, the efficacy and toxicity of new drugs are evaluated during non-clinical investigations that include pharmacokinetics (ADME, i.e., absorption, distribution, metabolism and excretion), pharmacodynamics and safety (safety pharmacology and toxicity) studies (FDA, 2013). The results of these studies are analyzed and extrapolated to predict efficacy, pharmacokinetics and potential adverse effects in humans. The phase I human study is typically performed in healthy volunteers that receive incremental doses of the drug to assess pharmacokinetics and tolerability and to reveal any potential adverse effects (Bass et al., 2009). Further efficacy and safety assessments are made in Phase II and III clinical trials if the test drug progresses through the phase I trial. Many adverse effects are only found in these clinical phases of drug development (Olson et al., 2000; Hartung and Zurlo, 2012; Hartung, 2013). The overall failure rate of candidate drugs in the clinical phase of drug development is higher than 80% depending on the area (Kola and Landis, 2004; Hornberg et al., 2014; Hay et al., 2014).

Most drug development is halted in the pre-clinical phase, and for every 20 compounds reaching the clinical trials, only one completes them (Hartung, 2013). As already reported, two of the major causes of attrition for market acceptance of new drugs are lack of efficacy and unwanted effects. Recent reports, including one from AstraZeneca, indicate that this trend has not changed over the last decade (Arrowsmith, 2011; Arrowsmith and Miller, 2013; Cook et al., 2014). Since preclinical drug development significantly relies on animal models to predict human effects, these data indicate that translation between species does not always work, i.e., animal data is not always predictive for humans (Olson et al., 2000; Ewart et al., 2014; Igarashi et al., 1995). The causes of attrition during development have induced pharmaceutical companies to invest enormous efforts to predict drug toxicity (and efficacy) very early during the drug development process, called “*frontloading of toxicity testing*” or “*early safety de-risking*” with the motto “*fail early, fail cheap*.”

In the last decades, the knowledge of molecular mechanisms of toxicity and the development of new, reliable *in vitro* screening methods have provided useful tools to screen new molecules very early in the drug development process. Almost all companies are using *in vitro* systems to evaluate possible genetic toxicity and cytotoxicity as well as screening to predict organ toxicity or to understand related molecular mechanisms (McKim, 2010; Horner et al., 2014).

## Question N° 1 – What are current initiatives?

In the period 2008–2010, the EU FP7 program supported the project START-UP^1^ (Scientific and Technological issues in 3Rs Alternatives Research in The process of drug development and Union Politics), with the intention to cover all the issues of the 3Rs-bottlenecks in pharmaceutical research and development. More than two hundred representatives from industry, academia and regulatory agencies had regular meetings, and while the concluding report is valuable, it is principally focused on 2Rs (reduction and refinement). The report offers little on how to predict the effect of a substance in the human organism with advanced cell technology. This report partially addresses this issue.

Projects aiming to review regulatory toxicology with overlapping tools and approaches include those supported by the Innovative Medicine Initiative^2^ (IMI), which is a public-private partnership between the European Union (represented by the European Commission) and the European pharmaceutical industry (represented by EFPIA, the European Federation of Pharmaceutical Industries and Associations) that was born with the idea of accelerating the drug approval process through better science. Its aim is to facilitate collaboration between universities, the pharmaceutical and other industries, small and medium-sized enterprises (SMEs), patient organisations and medicines regulators. IMI addresses both drug safety and efficacy; an example is the e-tox project^3^, which pools data from *in vivo* toxicity studies and links these data to clinical data (Cases et al., 2014).

EPAA^4^ (European Partnership for Alternative Approaches to Animal Testing) is a voluntary collaboration between the European Commission and companies that are committed to share knowledge and resources with the common goal of accelerating and accepting alternative methods in all different areas, i.e., not only toxicology. Under the umbrella of EPAA, there is a working group focused on understanding the potential of stem cells for safety assessment purposes^5^.

Momentum for change in Europe comes also from joint efforts between the industry and the regulators. This specific topic has been a matter of debate for several years already and workshops were organized to allow exchange of information between both parties and define a way forward. In October 2011, the Drug Industry Association (DIA) organized a workshop related to developmental and reproductive toxicity (DART): “*DART testing strategies for human pharmaceuticals- animal models vs. in-vitro approaches*” (van der Laan et al., 2012). The workshop aimed to discuss the value of rodent versus non-rodent species in the evaluation of human pharmaceuticals for their effects on embryo-foetal development. In addition, the workshop discussed the value of 3R methods to detect crucial developmental effects. Experience gained by pharmaceutical industry was shared with regulators and actions to further proceed in this matter were identified.

Within the Integrated Projects sponsored by FP7, the SEURAT-1 initiative^6^ is studying repeated dose toxicity. Co-funded also by Cosmetics Europe, it comprises six projects that aim to develop knowledge and technology building blocks for full replacement of the *in vivo* tests. SEURAT-1 is innovative as it integrates the efforts from 70 parties from many different sectors, even though the approach is still linked to the traditional idea of one endpoint – one method. One of the projects called ToxBank^7^ plans to establish a reference database containing information on both cell/tissue systems and chemicals, representing a potentially very useful platform for future expansions.

Other inputs arrive from national programs. Based on the UK House of Lords report in 2002 stating that “*the development of scientifically valid non-animal systems of research and testing is important, not just to improve animal welfare, but to provide substantial benefits for human health*”, the NC3Rs^8^, a national center for the Replacement, Refinement and Reduction of animals in research, was created with examples of significant contribution to the 3Rs whilst benefiting human health (Chapman et al., 2013).

## Question N° 2 – Is Toxicology in the 21^st^ Century (Tox21c) applicable beyond chemical risk assessment?

The Tox21c report has highlighted the limitations of animal models to predict complex toxicity outcome in humans. The NRC report in 2007 was directed to environmental chemicals, rather than drugs or food additives. ToxCast^9^ is a program within the US EPA than was generated to explore the 2007 NRC vision. It incorporates more than 700 diverse assay endpoints in a high throughput-screening (HTS) paradigm to assess the toxicity of thousands of chemicals. The assays include both cell-free and cellular systems, derived from multiple species and tissues. Tox-Cast aims to profile the bioactivity of all test chemicals in an unbiased way by testing the same concentration ranges and experimental protocols to each chemical, regardless of class. While this necessitates a broad, screening-level concentration range covering several orders of magnitude, it allows direct chemical-chemical comparison of potencies across all assays or subsets thereof. Phase I of ToxCast mainly studied pesticides, while Phase II covered a much broader area of chemical space (Kavlock et al., 2012). Compounds (plus associated human testing data) donated by six pharmaceutical companies (GSK, Hoffmann LaRoche, Sanofi-Aventis, Pfizer, Merck, Astellas), cosmetics ingredients (sponsored by L’Oréal) as well as some food additives are included in the list of tested substances. These compounds normally fall under the purview of the FDA, rather than EPA, so their inclusion in an otherwise environmentally-focused chemical set is a noteworthy opportunity for trans-disciplinary comparison. All data and results are made publically-available^3^ to allow modeling by interested research groups, use by stakeholders, and analysis in the context of external data on these same compounds. The program is ongoing, with major efforts directed toward computational methods to model these massive data (Reif et al., 2010), as well as extensions such as Tox21^™^ (an alliance between EPA, NIEHS/NTP, NIH/NCATS and FDA to screen over 8,000 unique substances across a subset of ~50 high-throughput assays) (Huang et al., 2014), and alternative *in vivo* models amenable to HTS (Truong et al., 2014).

Basing safety assessments on the mechanism of toxic action is at the core of Tox21c. With the progress in understanding mechanisms, toxicological research is increasingly moving to-wards mechanistically-based testing and assessment.

Pharmaceutical industry is interested in understanding the mechanism of action of a drug, either for efficacy or adverse effects. Re-analyzing the pathways leading to pathology is one basis for drug target identification. It should be noted that drug toxicity may include many disease patterns/pathologies produced by exaggerated or unselective pharmacology, i.e., untoward effects mediated through the targeted mechanism resulting in a potential PoT. This awareness can help in the compilation of all known pharmacologically relevant pathways and build a database similar to Effectopedia^10^ (a narrative descriptive database for pathways) or the Adverse Outcome Pathways (AOP) development by OECD^11^.

The Tox21c movement to pathways identification has clear benefits for the pharmaceutical industry: Such information supports species extrapolation (e.g., considering the fit of a given animal model), deviation from guideline studies and weighing evidence. Noteworthy, pathways can also be pathways for efficacy, i.e., any toxicity pathway can also be a pharmacological target as, for example, metabolomics and transcriptomics evaluating estrogen-regulated pathways in two human breast-cancer cell lines (Ramirez et al., 2013; Bouhifd et al., 2014).

The Human Toxome Knowledge-base (Kleensang et al., 2014) can become a point of reference for research and discussions with, e.g., regulators. It is also an opportunity for building computational networks, etc.

A hallmark of many of the Tox21c technologies is their holistic approach to information generation: ‘*omics* technologies are the prime examples in which the totality of genes, mRNA, microRNA, proteins, metabolites, etc. shall be assessed. Similarly, high-content imaging (HCI) determines a multitude of structural features and functions in an automated fashion. Last, but not least, even though HTS approaches, for example with an *in vitro* battery of tests, provide minor mechanistic understanding, the outcome is still very useful to immediately exclude substances with obvious toxicological liabilities. High content imaging (HCI) (van Vliet et al., 2014) already plays a role in investigative toxicology in pharmaceutical industry, i.e., to clarify toxic effects in guideline-driven studies or clinical trials and their relevance to the patient population. It is clearly an information-rich technique, but not really employed for PoT identification.

The concept of determining PoT using *in vitro* human-based systems and *in silico* methods is a logical progression to address toxic effects of chemicals based upon their mechanism of action/toxicity rather than merely dosing an animal with a chemical and looking at apical endpoints.

There are limits to the predictive value of any cell model to predict human responses, especially if the effects concerned are not cellular / organ responses but systemic, metabolic or even behavioral ones. The psychiatric sector is a self-evident example (though also the animal models of psychiatric diseases have their limitations); a drug must also reach a specific zone of the brain, so pharmacokinetic considerations have to be added; the activity of that substance may also activate the same receptor in another organ, which is difficult to predict *in vitro*. In addition, the quality of the cell culture procedure is very important, as all the details of the protocol are fundamental for precision and accuracy of the final outcome (Coecke et al., 2005).

Tox21c is mainly using human cell lines that presumably better represent PoT present in a human organism; animal cell lines should be used only if there is evidence of their relevance. Inherent limitations of cell culture models should be taken into account (Hartung, 2007). The use of human cells should be based on the demonstration of the *in vivo*-like functionality of the cells. Moreover pathway knowledge from pharmacology studies should be correlated with correspondent effects that may occur in humans before the beginning of clinical trials.

There has been an enormous improvement in cell culture methodologies. This includes 3D tissue-like cultures, cell co-cultures, perfusion cultures, various scaffolds, reactors, coatings and extracellular matrices, differentiation-inducing and maintaining factors, etc. (Hartung, 2014; Alépée et al., 2014). The US Human-on-a-chip program (Hartung and Zurlo, 2012; Andersen et al., 2014) aims to bring many of these components together in a microfluidic platform, even though similar approaches exist worldwide (Marx et al., 2012). These systems improve cell differentiation and functionality and hold the promise of being more predictive.

## Question N° 3 – Is the introduction of new strategies for regulatory safety assessment possible in the pharmaceutical industry

Regulatory safety assessments of pharmaceuticals are largely harmonized via the International Conference on Harmonization (ICH), which is responsible for issuing guidelines that represent recommendations, rather than protocols. ICH guidelines are complemented on a regional scale, for example, those that are drafted by the relevant Working Parties of the Committee for Human Medicinal Products (CHMP) at the European Medicines Agency (EMA). With respect to non-clinical testing requirements for human medicinal products, new *in vitro* methods have been accepted for regulatory use via multiple and flexible approaches, including formal validation, either as pivotal, supportive or as exploratory mechanistic studies, wherever applicable. Pharmaceutical regulators can drop redundant testing requirements. Indeed, data analysis following the publication of the concept paper on the need for revision of the EMA guideline on single-dose toxicity led to the complete removal of this guideline and its requirements, and thus a significant reduction in animal use (Chapman et al., 2010).

In theory the regulatory authorities in the pharmaceutical field would accept alternative strategies as a matter of course when they are considered qualified for a specific context of use. However, there is no definition of what “good” means and how much evidence should be provided to demonstrate effectiveness and safety. The general tendency in toxicology is to introduce new technologies without quitting the old methods, which always stay in place. Historically, performance of 3R methods has been tested against the *in vivo* animal data, which can entail problems as new methods have the ambition to improve the relevance and specificity to humans. The principle overcomes the idea of one endpoint being replaced by one or a set of *in vitro* methods, and aims rather to elucidate the global assessment of the impact that a drug may have on human organisms, with a full risk assessment. In this sense there is no parallel with the current procedure, which is well established and difficult to modify.

In the EU, Directive 2010/63/EU on the protection of animals used for scientific purposes, which is fully applicable to regulatory testing of human and veterinary medicinal products, unambiguously fosters the application of the principle of the 3Rs and requires that a 3R method be used whenever that method is recognized under the legislation of the Union, and this has many implications in the pharmaceutical world. In October 2010, the EMA set up a Joint ad hoc Expert Group (JEG 3Rs) aiming to improve and foster the application of the 3Rs to regulatory testing of medicinal products (EMA, 2011, 2014). This group advises the EMA scientific committees on all matters related to the use of animals in regulatory testing of medicinal products. It is composed of experts from the EMA scientific committees and the working parties to which animal testing is relevant and works in close cooperation with the European Directorate for the Quality of Medicines & HealthCare (EDQM) and the European Union Reference Laboratory for Alternatives to Animal Testing (EURL-ECVAM). In addition, JEG 3Rs coordinates responses to requests from EURL-EC-VAM for preliminary analysis of regulatory relevance of new alternative methods. A draft guideline on regulatory acceptance of 3Rs testing approaches has recently concluded public consultation and is now being amended. This guideline describes the process for submission and evaluation of a proposal for regulatory acceptance of 3R testing approaches as well as scientific and technical criteria for qualification, and it anchors the pathway for regulatory acceptance within the EMA Scientific Advice Working Party (SAWP) through its procedure for qualification of novel methodologies (Manolis et al., 2011).

In the USA, advancing regulatory sciences and applying new technologies for consumer protection is a goal and FDA wants to be involved in the validation (qualification) of new methods. There are considerations for animal welfare as well. FDA has developed a strategic plan for regulatory science by developing new tools, standards and approaches to assess the safety, efficacy, quality and performance of FDA-regulated products^12^. The FDA’s strategic plan has the vision: “speed innovation, improving regulatory decision” and includes eight priority areas.

1. Modernize Toxicology to Enhance Product Safety
2. Stimulate Innovation in Clinical Evaluations and Personalized Medicine to Improve Product Development and Patient Outcomes
3. Support New Approaches to Improve Product Manufacturing and Quality
4. Ensure FDA Readiness to Evaluate Innovative Emerging Technologies
5. Harness Diverse Data through Information Sciences to Improve Health Outcomes
6. Implement a New Prevention-Focused Food Safety System to Protect Public Health
7. Facilitate Development of Medical Countermeasures to Protect Against Threats to U.S. and Global Health and Security
8. Strengthen Social and Behavioral Science to Help Consumers and Professionals Make Informed Decisions about Regulated Products

A toxicology working group in FDA is raising the profile of toxicology. Active collaboration with EPA and NIH in the Tox21 alliance promotes Tox21c implementation via HTS, which is seen as a driving force to protect and promote human health. The National Center for Toxicological Research^13^ (NCTR) in Jefferson, Arkansas brings scientists together and has active programs to promote toxicology of the different products under FDA mandate. NIH and FDA give grants for improving regulatory approaches. Various Law School initiatives are helping to overcome legal hurdles to using test-systems. Overall, it is remarkable how FDA is promoting new developments. Two workshops hosted by FDA on new approaches are good examples (Stallman Brown et al., 2012; Andersen et al., 2014).

## Question N° 4 – Is the introduction of new strategies for regulatory safety assessment useful for food safety assessment?

The food industry differs from the pharmaceutical industry in that its primary aim is not to cure diseases but to improve food quality and taste as well as providing adequate nutrition to help in the prevention of the occurrence of diseases. Importantly and in contrast to pharmaceuticals, that in most case target a specific population group, food is for the general population, including babies, pregnant women and the elderly. Unlike pharmaceuticals, for which a benefit/risk evaluation is systematically considered, a benefit/risk approach is rarely used for ingredients voluntarily added to regular foodstuffs, since typically no risk is accepted for the general population.

In the EU, efforts to regulate direct food additives began in the 1960s with a directive listing food colors followed by preservatives (1964), antioxidants (1970) and emulsifiers, stabilizers, thickeners and gelling agents (1974) (van der Meulen, 2013). The first horizontal harmonization between EU member states dates from 1988 (Directive 89/107/EEC on the approximation of the laws of the Member States concerning food additives authorized for use in foodstuffs intended for human consumption). From 2001, the scientific evaluation of food additives has been framed by the European Scientific Committee on Food (SCF, former EFSA). In the EU, since the entry into force of Regulation EC 258/97 on novel foods and novel food ingredients, all new ingredients that were not consumed to a significant degree before May 1997 must undergo a novel food approval and appropriate toxicity testing.

In the US, the first initiatives to regulate food chemicals date from the Pure Food and Drugs Act of 1906 that already acknowledged the concerns about chemicals in food and started to establish a framework for FDA’s regulation on food colors. The main distinction of the US FDA from the other evaluation systems is the unique exemption from premarket approval requirements for uses of food ingredients that are generally recognized as safe (GRAS) by qualified experts (GRAS exemption).

Along with the continuous EU Novel Food registration process for new ingredients to enter the EU market, the European Union has now enforced a cyclic re-evaluation of food additives and set an expiry period of 2020 for the authorization of all food additives (Regulation EC 1333/2008) that have been in use for decades and have not undergone a safety evaluation under the current toxicity testing standards. However, a recent report focused on the specific US situation with emphasis on the FDA GRAS process, indicated that 80% of toxicological data are missing or not publically available (Neltner et al., 2013). While a significant history of safe usage exists for most of these food additives or ingredients, lack of detailed data still can create some discomfort as these substances are consumed in sometimes large quantities over many years. On the other hand, it is quite understandable that a substance that is a nutritional component naturally occurring in the diet and/or has already a proven history of safe use in foods and is recognized as GRAS and has not triggered any alerts as to its safety should not have to undergo the complete battery of toxicity testing to the same standards of drug development. This would for instance be the case for many ingredients or additives like vitamin C (i.e., used as antioxidant), starches and fibers (i.e., used as thickeners or emulsifiers). Furthermore, only very few entirely new food additives have been developed and approved since the 1960s.

Toxicity of functional ingredients is not always assessed at the highest maximum tolerated doses in standard animal toxicity studies. Doses ranging up to about 10-fold the human consumption levels can be employed in the context of ingredients having a nutritional function. For example, in case of dietary fibers the level tested in chronic toxicity studies can be about ten-times the dose in humans in order to avoid nutritional imbalance, which would trigger unspecific nutritional effects that are not linked to any intrinsic toxicity. In contrast, food additives and other food chemicals are most often tested at much higher levels in order to derive a health-based guidance value (i.e., Acceptable Daily Intake) that is at least 100-times lower than (or even lower) the highest dose shown not to induce adverse effects. The magnitude of exposure in animal studies can be much higher if we compare to ingestion levels resulting from the approved levels in single food stuffs consumed daily. (Maximum) Acceptable or Tolerable Daily Intake (ADI/MTDI) is derived from the generated toxicity dataset. Besides the hazard identification and characterization through toxicity testing, another crucial step in the overall risk assessment process of functional ingredients or food additives is the exposure assessment based on maximum use-levels of the ingredient, maximized/worst case dietary intake scenarios (i.e., 90–95^th^ percentile food consumption from dietary surveys) and toxicokinetics.

There used to be little harmonization in the food area, so when a new ingredient was introduced in a country it had to comply with the regulation and testing requirements of that area. This can explain the historical divergences in and above-mentioned discomfort about the approaches used for instance within Europe and in the US. But, nowadays, increasing harmonization is observed, since EFSA collaborates more frequently with JECFA (and FDA) on food safety-related topics. In most cases, recent guidelines for submission for regulatory approval will require the same minimum standard tests to be conducted. In some cases, the differences might also lie in the expert interpretation of the outcome of the toxicity studies with some expert committees requesting complementary toxicity tests while others would consider the submitted dataset satisfactory for regulatory approval. Specific guidance for testing is available from EFSA’s guidance documents, JECFA’s Environmental Health Criteria (240) and FDA’s Redbook (see Tab. 1). In most cases standard testing for new food ingredients and additives complies with OECD testing, which allows also achieving harmonization in the quality standards for testing. The first steps generally include standard pre-clinical *in vitro* (i.e., genotoxicity, digestibility/metabolism) combined with *in vivo* studies (i.e., subchronic, reproductive toxicity). In the case of functional ingredients, a second step may involve investigations in human volunteers. Other areas like industrial and agrochemicals do not have access to human testing. Functional foods and ingredients (also termed nutraceuticals) tend to be perceived as more similar to drugs but in comparison to drugs they are less studied, though as highlighted above guidance from the authorities on how to provide the minimal datasets for regulatory approval is now well established.

Risk-benefit considerations may be used in exceptional cases, for instance in case of food ingredients used in particular diseased groups that require specific nutrition and product formulation. Minor or transient effects (i.e., tolerance) could be considered acceptable if the critical goal is to restore and maintain an adequate nutrition status (i.e., cancer patients). Other industries also do not perform risk-benefit analysis.

The current legal requirements and guidance for evaluation of food ingredients still have an important focus on traditional animal toxicity data (i.e., 90-day feeding studies) (EFSA, 2012). This will undoubtedly hamper the transition to new alternative toxicity testing methodologies in that area. At the same time, it should be acknowledged that important challenges remain in the context of the scientific relevance, the successful validation and the implementation of alternative testing strategies for food ingredients. This includes, amongst others, the complexity of the food matrices into which a food ingredient is introduced (i.e., not a single substance but mixture of ingredients within a food matrix), multicellular and inter-organ cooperation in digestion and metabolism (ADME), representativeness and availability of models to cover all life-stages, inter-species difference of the GI system, the ability to make a clear distinction between a beneficial physiological response to a nutritional substance and possible adverse outcome pathways. Recent efforts promoted for instance at EFSA level, tend to demonstrate the paradigm shift for food ingredients and chemical risk assessment since alternative testing and ITS approaches are more and more used on a case by case basis to fill in the risk assessment data gaps, to bridge the minimal animal dataset to the human situation or to prioritize for additional animal testing (i.e., QSARs, read-across, threshold of toxicological concern) (EFSA, 2014). There is yet little correlation between the real willingness to change and the number of official positions that can be issued. For example, to determine the potential risk for genotoxicity EFSA has produced a guideline that suggests a stepwise approach (EFSA, 2011). First, *in vitro* tests are performed to determine genotoxicity, and only if some of these tests are positive *in vivo* studies will be performed. However, during the overall risk assessment, *in vivo* studies are frequently requested. Foods and food ingredients are less in the focus of animal welfare organizations, but pressures are similar to other industries. EFSA has for instance given a lot of emphasis to animal welfare considerations (EFSA, 2009) and actively encourages petitioners to use alternative tests when submitting dossiers for food additives evaluation.

## Discussion

The real question is whether the actual procedures are satisfactory and in case the answer is negative, if there is something different that can be better, keeping in mind that the ultimate goal for all sectors is human prediction.

In 2000, Olson et al. published an interesting study titled: “Concordance of the toxicity of pharmaceuticals in humans and in animals,” in which the limits of selected animal models are presented with statistics and scientific evidence. Pharmaceutical companies are well aware of the situation, and *in vitro* strategies are more and more in use during the first steps of lead identification / lead optimization. The example of the Olson et al. paper should be extended to retrospectively compare results from human studies with animal data. Several such data sets have been collected in the course of the IMI eTOX project, but they have not (yet) been made available for the public. More studies like this should be available to the scientific community (Leist et al., 2014) to have new tools for comparing animal results with the effects in humans with the aim to better understand the relevance of the new techniques; to this effect it should be noted that some recent examples are emerging (Ewart et al., 2014; Keating et al., 2014; Valentin et al., 2013). Moreover, this type of approach may help to distinguish between true side effects of the new drug and the unforeseen adverse event that derives from excess pharmacology. Other variables should be included in the human prediction, like the human population variability, life stage, presence of other diseases, etc. Final risk assessment should mainly consider a probabilistic approach with indepth statistical evaluation. In recent years, the movement on Evidence Based Toxicology (EBT, http://www.ebtox.com) has found proponents in both the US and the EU, aiming to improve the validity of toxicological assessments by systematically implementing transparency, objectivity and consistency in the evaluation. EBT may help in designing the strategy before the execution of the tests, by making a conscious, rational decision about what to include and what to exclude with a consequently increased acceptance of the outcome.

**Fig 1:**
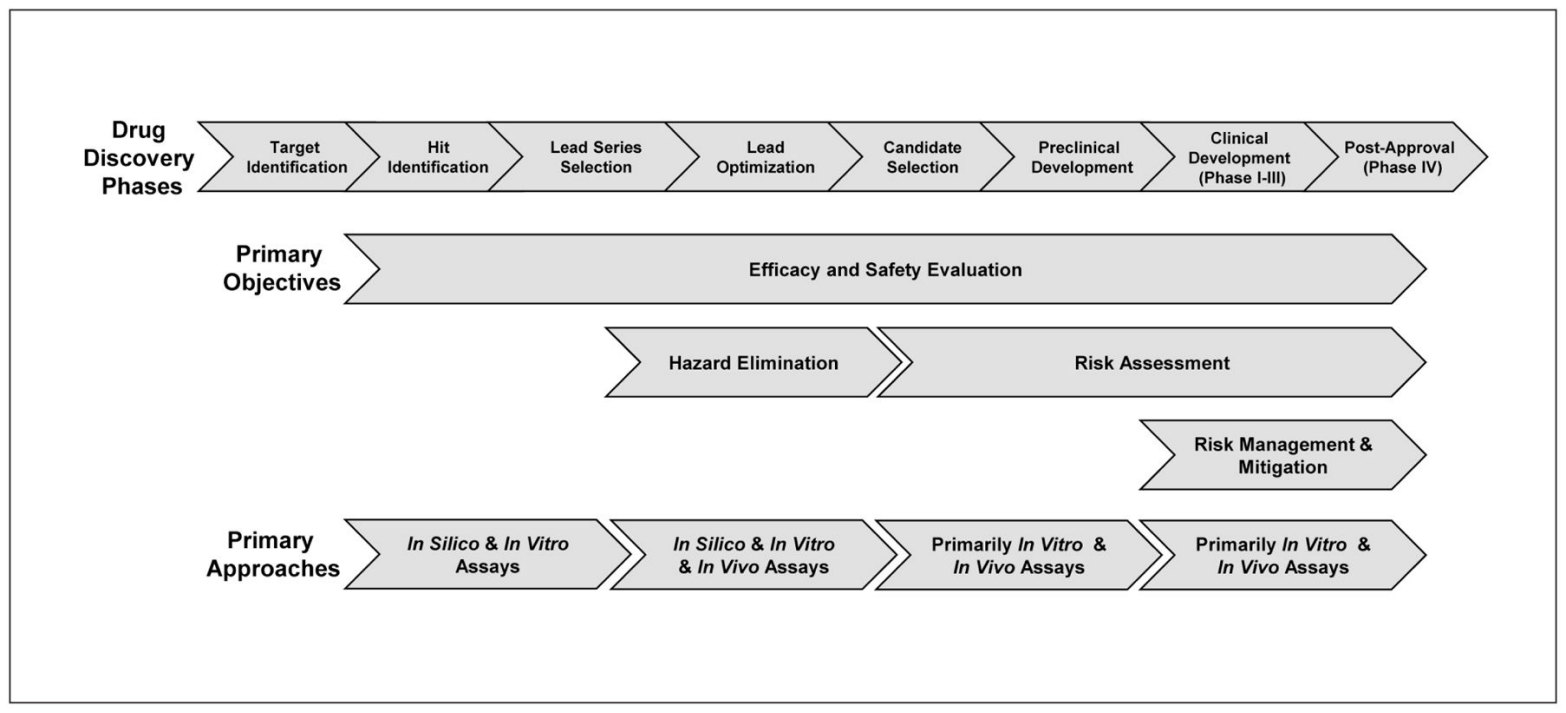
Alignment of assays to the pharmaceutical drug discovery and development process Drug discovery phases start with target identification, where many chemicals are tested with screening methods and move towards the assessment of fewer and fewer substances with an increasing knowledge deriving from more and more complex methods until the ideal case when no adverse effects are detected in clinical phases. *In silico* and *in vitro* methods play an important role at the beginning of the process, when screening tests give the opportunity for a rapid and convenient assessment, and along the whole life of drug development to elucidate the precise mechanism of action.

Investments to understand toxicological conditions are made by implementing and validating *in vitro* screening methods that are less resource- and compound-dependent and generally cheaper and faster to assess toxic liabilities. Identifying the molecular mechanisms of toxicity may help in the discovery of new endpoints and biomarkers to be used in non-clinical and clinical studies, plus in extrapolating toxicity from animals to humans. Alternative strategies including *in vitro* methods and modeling are already widely used in the early phase of drug development, with the aim to identify leads and to elucidate the Mechanism of Action (MoA). Alignment of methods along the drug development process (Fig. 1) may dramatically benefit from a more holistic approach, as proposed by the Tox21c paradigm.

Acceptance of alternative strategies by regulators in both the pharmaceuticals and food field is not a hurdle when proven scientifically qualified for the specific context of use. However, the general tendency in toxicology is to introduce new methods that add knowledge without eliminating the old methods. For example, introduction of ICH guideline S5(R2) on “Detection of toxicity to reproduction for medicinal products & toxicity to male fertility”^14^ seems to open to new strategies stating that: “*No guideline can provide sufficient information to cover all possible cases, all persons involved should be willing to discuss and consider variations in test strategy according to the state of the art and ethical standards in human and animal experimentation*.” However, the whole guideline describes the requirements for a good animal test. Section 2.2 considers the possibility of applying *in vitro* methods, but the text is clear in saying that this approach may only add information, but cannot be a standalone approach: “*2.2. Other test systems: Other test systems are considered to be any developing mammalian and non-mammalian cell systems, tissues, organs, or organism cultures developing independently* in vitro *or* in vivo*. Integrated with whole animal studies either for priority selection within homologous series or as secondary investigations to elucidate mechanisms of action, these systems can provide invaluable information and, indirectly, reduce the numbers of animals used in experimentation. However, they lack the complexity of the developmental processes and the dynamic interchange between the maternal and the developing organisms. These systems cannot provide assurance of the absence of effect nor provide perspective in respect of risk/exposure. In short, there are no alternative test systems to whole animals currently available for reproduction toxicity testing with the aims set out in the introduction*.”

The high number of failures of drugs during clinical development speaks out for an innovative approach to access toxicity and pharmacology information in a faster and more reliable way. The paradigm shift that was described in the Tox21c report may provide new tools by substituting traditional animal tests with *in vitro* tests on human derived cell systems organized in advanced strategies. The time is ripe to foster this development as well as a change in mind set to minimize the risk of unforeseen data from animal tests as quickly as possible (see e.g., Evans, 2014).

Risk assessors are transitioning from a checklist for testing to a more strategic effort to understand the mechanisms of action of a given compound or hazard. Indeed, in the last two decades, this has increasingly enhanced the capacity to understand 1) mechanisms leading to a toxicity findings and 2) its relevance to humans. These are two questions that are addressed by introducing relevant and specific biomarkers and endpoints and by complementing the conventional regulatory package with investigative/mechanistic data obtained both *in vivo* and *in vitro*. Mapping the human toxome (Bouhifd et al., 2013), i.e., the attempt to establish a knowledge base of the molecularly defined PoT, is an effort to do this systemically. Pharmaceutical industries are increasingly showing interest, while notably the food area is not very much involved at this moment. On the other hand, detecting target organ toxicity continues to be a challenge (Cook et al., 2014; Horner et al., 2014). New methodologies, such as the human-on-a-chip approach (the combination of different three-dimensional [stem] cell-based organ equivalents connected with microfluidics), represent novel tools for addressing multi-organ interactions (Hartung and Zurlo, 2012).

An integrated testing strategy (ITS) approach, in which a series of mechanism-based tests is employed, is a likely scenario for future regulatory safety testing. This approach is appropriate, for example, when one test cannot detect all possible mechanisms of action or all types of substances of interest (Hartung et al., 2013). There is a connection between PoT-based approaches and ITS; since several pathways or key events are typically involved in a toxic effect (hazard), testing needs to reflect them, often by combining several tests. The pharmaceutical industry is employing many sophisticated testing strategies, but there is no harmonization between companies and this use is different to the envisaged ITS for testing batteries in regulatory use (Rovida et al., 2015). This is a matter of terminology: an ITS does not simply involve combining many tests, but also their integrated interpretation and validation of results. The pharmaceutical industry has some interests in ITS, but there is some fear they might become rigid though the need for formalization is recognized.

The drug approval process has relied on animal safety data sets to a large extent. However, the experience gained with this approach is too solid to be abandoned in a short time and any change should be introduced in a stepwise mode. The main hurdle derives from the effort that is required. Regulators and national institutes should subsidize it in the interest of the general population. For example, some initiatives should include funding new studies that apply advanced techniques, fast track evaluation of dossiers that include the double assessment, fee reduction, and so on. Along these lines, in the field of cardiac drug safety, there are on-going initiatives aiming to define a new paradigm in which proarrhythmic risk would be primarily assessed using non-clinical *in vitro* and *in silico* human models based on solid mechanistic considerations of torsades de pointes proarrhythmia (Sager et al., 2014).

New *in vitro* technologies should be accepted following a tailored qualification that includes fit for purpose assessment, reproducibility, mechanistic validation and demonstration of relevance. In fact, the classical validation paradigm is not appropriate for new methods. Quality assurance should have the highest priority for strengthening the scientific basis for evaluation. Scientific discussion on this topic has just begun and full applicability of this concept is not yet mature. However, more efforts should be directed towards this goal.

An interesting approach is the safe harbor concept (Lesko et al., 2003), when two strategies are applied in parallel and compared. Submitted data from new approaches are not immediately used for decision-making; rather the concept of Safe Harbor asks that for a certain period, the new 3R method is run in parallel with the conventional test, in order to collect information that may then help to make the ultimate decision for regulatory acceptance of the new method. Long-term experience will demonstrate whether the new strategy is more effective. This gives the regulators insight into its usefulness and could support the fit-for purpose qualification.

To cope with the new challenge, it is of utmost priority to collect all available information in an inventory that must be publicly accessible to some extent, even though protecting confidential information. More cross talk between the US and EU should be encouraged.

As requested by Tox21c, toxicology should be based on the evaluation of the cell toxicity pathways that need to be carefully mapped. The project is ambitious and huge and thus a systematic approach for identification of the toxicity pathways should be established. A starting point may be the selection of priority pathways with substances that work through well-known pathways. The project should be developed in many research centers and therefore a centralized institution should coordinate the activities and collect the data that must be organized in a suitable database. The human toxome project is ongoing in the area of chemical risk assessment. Enlargement to include needs of the pharmaceutical and food industries should not be impossible and *vice versa*.

From this point of view, the pharmaceutical approach is very much different compared to the evaluation of chemicals, because human data are available and patient doses known. The food industry often has few human safety data available. However, such data exist for non-desired food ingredients, such as pesticides, where realistic modeling of exposure can be obtained by measuring residues in crops and other potential sources along the food chain. In addition to that, the new paradigm may also accelerate the approval process, hopefully, with fewer side effects on humans. On the reverse side, there are probably many effective drugs that do not enter the clinical phase only because of negative effects on animals during the non-clinical phase.

## Conclusions

The application of Tox21c approaches may aid the safety prediction for drugs and novel food. The process is very complex and needs further work, also considering that implementation is not through replacement of individual patches.

Starting from what is available today, retrospective analyses of new human data may help in the definition of the new strategy. Currently, new *in vitro* methods are validated against animal models; However, this approach will not help to improve the prediction of the effects of a substance in the human organism in case the animal model is biased.

The pharmaceutical industry has already welcomed the mechanistic thinking, but there is a gap between research and regulatory acceptance. The latter must be open to 1) evaluation of novel methods for human relevance, 2) not adding new methods as an additional layer atop traditional methods (after a process to assess usefulness of both). Solid data are needed before a paradigm shift can be realized.

The paradigm shift must be introduced gradually. Accepting new methods is not straightforward since they will never replace a traditional one directly; most of the time, a new method will refine the strategy, which includes the abandoning of earlier components, and in the future this may lead to a reduced need for animal data. The new technologies, including omics, computer modelling, mechanistic approaches, etc. are definitely underexploited for regulatory purposes, even though the majority of the novel methodologies are already applied in screening during drug discovery and as such avoid bad candidates making it to regulatory testing.

## Figures and Tables

**Tab. 1:** Main guidance documents for toxicity testing of food ingredients and additives

Food Safety Authority	Core guidance document for toxicity testing	Link
**EFSA**	Guidance for submission for food additive evaluations	http://www.efsa.europa.eu/en/efsajournal/pub/2760.htm
**EFSA**	Guidance on conducting repeated-dose 90-day oral toxicity study in rodents on whole food/feed	http://www.efsa.europa.eu/en/efsajournal/pub/2438.htm
**FDA**	Toxicological Principles for the Safety Assessment of Food Ingredients Redbook 2000	http://www.fda.gov/downloads/Food/GuidanceRegulation/UCM222779.pdf
**WHO**	EHC 240: Principles and methods for risk assessment of chemicals in food	http://www.who.int/ipcs/food/principles/en/index1.html
**Food Safety Commission (Japan)**	Guidelines for Assessment of the Effect of Foods on Health for Food Additives (Translated from Japanese)	http://www.fsc.go.jp/senmon/tenkabutu/tenkabutu-hyouka-shishin.pdf

## Footnotes

1 http://cordis.europa.eu/project/rcn/88173_en.html

2 http://www.imi.europa.eu

3 http://www.etoxproject.eu

4 http://ec.europa.eu/enterprise/epaa/

5 http://ec.europa.eu/enterprise/epaa/platform-science/stem-cells/stem_cells_en.htm

6 http://www.seurat-1.eu

7 http://toxbank.net/

8 http://www.nc3rs.org.uk

9 http://www.epa.gov/ncct/toxcast/

10 http://www.effectopedia.org

11 http://www.oecd.org/chemicalsafety/testing/adverse-outcome-pathways-molecular-screening-and-toxicogenomics.htm

12 http://www.fda.gov/downloads/scienceresearch/specialtopics/regulatoryscience/ucm268225.pdf

13 http://www.fda.gov/AboutFDA/CentersOffices/OC/OfficeofScientificandMedicalPrograms/NCTR/

14 http://www.ich.org/products/guidelines/safety/article/safety-guidelines.html (Guideline currently under revision)
